# Synthesis, Quality Control and Stability Studies of 2-[^18^F]Fluoro-2-Deoxy-D-Glucose(^18^F-FDG) at Different Conditions of Temperature by Physicochemical and Microbiological Assays

**Published:** 2017

**Authors:** Siyavash Rahmani, Soraya Shahhoseini, Reza Mohamadi, Mostafa Vojdani

**Affiliations:** a *PET/CT Unit, Ferdous Nuclear Medicine Center, Dr Masih Daneshvari Hospital, Shahid Beheshti University of Medical Sciences, Tehran, Iran.*; b *Pharmaceutical Chemistry Department, School of Pharmacy, Shahid Beheshti University of Medical Sciences, Tehran, Iran.*

**Keywords:** Fludeoxyglucose(18F-FDG), PET/CT, Quality control, Stability, Radiopharmaceutical

## Abstract

The introduction of 2-[^18^F] fluor-2-deoxy-D-glucose (^18^FDG) has provided a valuable tool for the study of glucose metabolism in both normal and diseased tissue in conjunction with positron emission tomography (PET). ^18^FDG is the most important radiopharmaceutical to be used in Nuclear Medicine for studying the brain, heart and tumor. The advancement in synthesis and quality control of ^18^FDG and its approval by US FDA are main reasons for increasing clinical application of ^18^FDG over the last 20 years. In this manuscript we explain the synthesis, quality control and stability studies of ^18^FDG (evaluate the physicochemical and microbiological stability of ^18^FDG, stored at room temperature (18 - 23 °C), and 35 - 40 °C, at different time intervals). We investigated how the influence of environmental factors in different lengths of time, alters the quality of this radiopharmaceutical. The pH, radionuclidic identity and purity, radiochemical identity and purity, chemical purity, bacterial endotoxins and sterility of ^18^FDG were evaluated according to the European Pharmacopoeia 7ed. analytical methods and acceptance criteria. The results suggest that under experimental conditions^ 18^FDG has physicochemical and microbiological stability up to 10 h after the end of synthesis.

## Introduction

**Scheme1:**
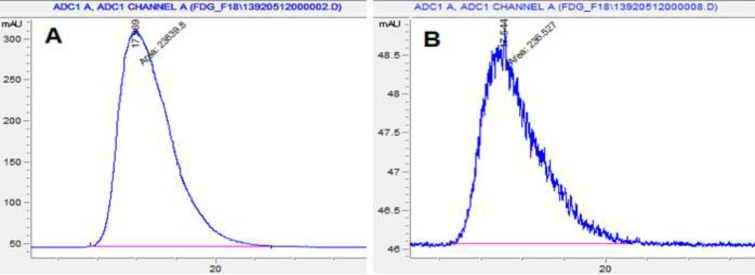
Synthesis of ^18^F-FDG by nucleophilic substitution

Cancerous cells have a high metabolic rate for glucose and accumulate glucose at a higher rate than normal cells in the body. The most useful target in the clinical practice of Positron Emission Tomography (PET) to date has been the increased glucose metabolism present in most cancers. 2-[^18^F]fluoro-2-deoxy-D-glucose (^18^FDG) was developed as a glucose analog to study the initial steps of glucose metabolism in the brain. ^18^FDG is the most widely used radiopharmaceutical in the expanding medical imaging field of Positron Emission Tomography (PET) (1). The chemical structure of ^18^FDG is the same as glucose except that the hydroxyl group on the 2-carbon of a glucose molecule is replaced by a fluoride atom with the retention of stereo-configuration of glucose. ^18^FDG is transported into cells by glucose transporters and phosphorylated by hexokinase to ^18^FDG-6-phosphate. Since molecular alterations in glucose metabolism of cancer cells such as overexpression of facilitative glucose transporters and hexokinase, the ^18^F-FDG transport into cancer cells and phosphorylation increases.^ 18^FDG-6-phosphate would not further metabolize after this step, retained and can be imaged by PET. Whole body PET imaging with ^18^FDG measures glucose metabolism in all organ systems with a single examination. Since cancer is a systemic disease, FDG-PET allows the early detection and quantification of metastasis. Therefore, it has found applications in the diagnosis, staging, and restaging of several clinical conditions such as lung cancer, colorectal cancer, lymphoma, melanoma, head and neck cancer, brain and breast cancer (2). Clinical applications of FDG-PET are also found in neurology, cardiology and inflammation/infection. The synthesis of ^18^F-FDG begins with the production of [^18^F]fluoride in a cyclotron, typically in a target chamber containing [^18^O]H_2_O, followed by a nucleophilic reaction with 1,3,4,6-tetra-O-acetyl-2-O-trifluoromethanesulfonyl-β-Dmannopyranose (mannose triflate) (3), and subsequent de-blocking of the protecting groups (acetyl), resulting in FDG formation (Scheme1). In general, the synthesis is performed in an automated synthesis module within a hot cell while the air environment is being controlled. The precursor molecule for the radiochemical synthesis of ^18^FDG (mannose triflate) is a sugar molecule containing a suitable leaving group (trifluoromethanesulfonyl) for a facile nucleophilic reaction at carbon 2 in the molecule, while the other four potential reaction sites are blocked with protecting groups (tetra-acetyl).

**Figure 1.**
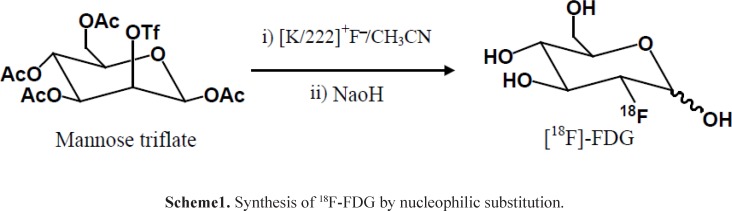
MCA Chromatograms of ^18^F-FDG after synthesis (A) and 10 h later (B) at r.t.

**Figure 2:**
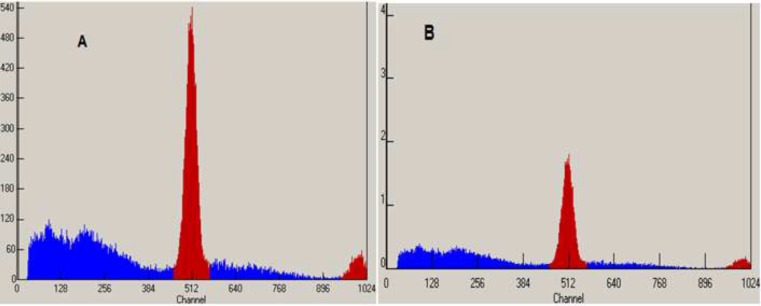
MCA Chromatograms of ^18^F-FDG after synthesis (A) and 10h later (B) at 35 - 40 ºC

**Figure 3:**
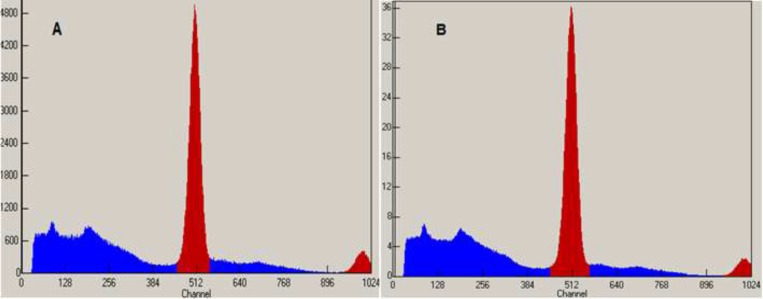
TLC Scanner Chromatograms of ^18^F-FDG after synthesis (A) and 10 h later (B) at r.t.

**Figure 4:**
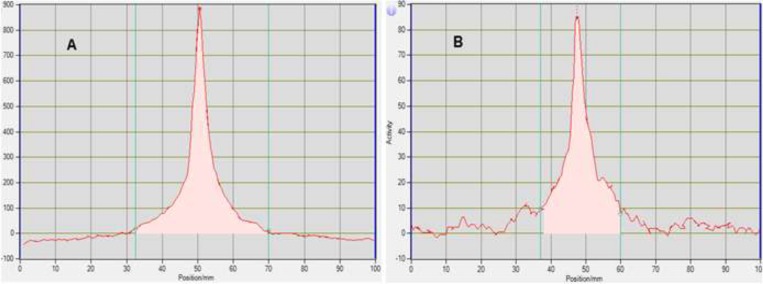
TLC Scanner Chromatograms of ^18^F-FDG after synthesis (A) and 10 h later (B) at 35 - 40 ºC

**Figure 5:**
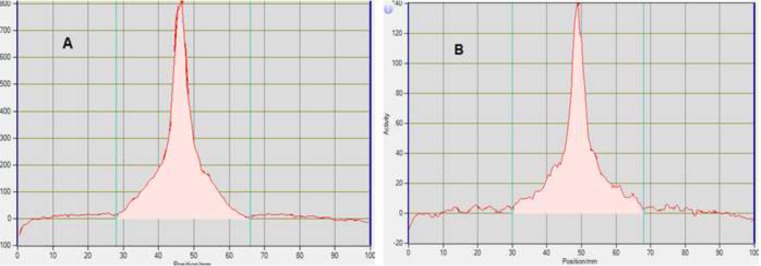
HPLC Chromatograms of ^18^FDG after synthesis (A) and 10 h later (B) at r.t.

**Figure 6:**
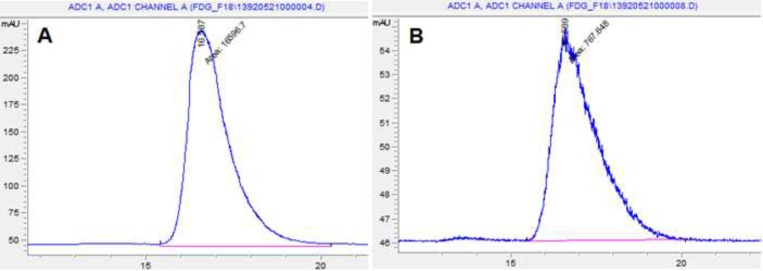
HPLC Chromatograms of ^18^F-FDG after synthesis (A) and 10 h later (B) at 35 - 40 ºC

**Table 1:** Specifications for ^18^F-FDG (EP 7ed, 2012)

**Test**	**Method**	**Specification**
**pH**	pH paper	pH: 4.5 - 8.5
**Radionuclidic purity**	Half life Determination	T_1/2_ : 105-115 min T_1/2_ = (0.693×t)/Ln(A_0_/A_t_)
	Gamma spectrometry (MCA)	There shouldn’t be peak observations except 0.511MeV and 1.024MeV energy levels
**Radiochemical purity and identity**	HPLC analytical	^ 18^FDG: 95% ^ 18^FDG + ^18^FDM: 95% ^ 18^FDM/(^18^FDG + ^18^FDM): 10% ^ 18^F + ^18^FDG derivatives: 5%
	TLC	%^18^F- ^18^FDG: > 95% %^18^F- fluorine: < 5% R_f_ FDG standard 0.05
**Chemical purity**	GC	Ethanol : 50 mg/V(mL)
	GC	Acetonitril : 4.1 mg/V(mL)
	TLC	Kryptofix 222 : 2.2 mg/V(mL)
	HPLC	FDG, FDM and glucose Peak areas obtained with ^18^FDG solution, shouldn’t be two times greater than area of the ^18^FDG peak obtained with the reference solution, ^18^FDG: 0.5mg/V(mL)
**Pyrogen test**	LAL tester	< 175 EU/V(mL)

**Table 2:** Results of ^18^F-FDG assays at different time intervals at room temperature

***Parameters***	***Results***					
	**0h**	**2h**	**4h**	**6h**	**8h**	**10h**
**Half-life(min)**	109.5	109.7	108.8	109.8	109.3	108.7
**Radionuclidic identity(keV)**	511 ± 1	513 ± 1	511 ± 1	512 ± 1	510 ± 1	511 ± 1
**Radiochemical identity (R**_f_ **)**	0.5	0.5	0.49	0.5	0.49	0.48
**Radiochemical purity**	97	98	96	98	97	98
**pH**	5.5±0.5	-	-	-	-	5.5±0.5
**Chemical purity**	Kryptofix: 2.2 mg/3mL	-	-	-	-	Kryptofix: 2.2 mg/3mL
	Ethanol: 0.014 mg/3mL	-	-	-	-	Ethanol: 0.012 mg/3mL
	Acetonitrile: 0.0019 mg/3mL					Acetonitrile: 0.0017 mg/3mL
**Bacterial endotoxins**	<1 EU/V(mL)	-	-	-	-	<1 EU/V(mL)
**Sterility**	Sterile	-	-	-	-	Sterile

**Table 3:** Results of ^18^F-FDG assays at different time intervals at 35 - 40 ºC.

***Parameters***	**Results**					
	**0h**	**2h**	**4h**	**6h**	**8h**	**10h**
**Half-life(min)**	109.3	108.5	109.2	109.5	109.9	109.6
**Radionuclidic identity(keV)**	513 ± 1	511 ± 1	510 ± 1	511 ± 1	514 ± 1	512 ± 1
**Radiochemical identity (R**_f_ **)**	0.48	0.49	0.49	0.49	0.49	0.49
**Radiochemical purity**	98	98	99	98	96	98
**pH**	5.5 ± 0.5	-	-	-	-	5.5 ± 0.5
**Chemical purity**	Kryptofix: 2.2 mg/3 mL	-	-	-	-	Kryptofix: 2.2 mg/3mL
	Ethanol: 0.0034 mg/3mL	-	-	-	-	Ethanol: 0.0029 mg/3mL
	Acetonitrile: 0.0021 mg/3mL					Acetonitrile: 0.0016 mg/3mL
**Bacterial endotoxins**	<1 EU/V(mL)	-	-	-	-	<1 EU/V(mL)
**Sterility**	Sterile	-	-	-	-	Sterile

The increasing demand for ^18^FDG requires an increase in production without a decrease in quality (4). It has been reported that, regardless of the radioactive decay, ^18^FDG decomposes in-vitro, resulting in the degradation of the radiochemical purity with time (1, 5). Because of 110 min half-life of ^18^F, it is possible to deliver ^18^FDG to PET imaging centers distant from cyclotron and production facilities. Because of different weather conditions in Iran, the aim of this study was to synthesis, quality control and evaluate the physicochemical and microbiological stability of ^18^FDG (pH, radionuclidic and radiochemical identity and purity, chemical purity, bacterial endotoxins and sterility), stored at room temperature (18 – 23 °C) and (35 – 40 °C) at different time intervals (6).

The first PET facility (including cyclotron, radiopharmacy, and PET/CT camera) in Iran was installed at Ferdous Nuclear Medicine Center, Dr Masih Daneshvari Hospital, Shahid Beheshti University of Medical Sciences, Tehran. 

## Materials and methods

All chemicals, reagent kits, cassette were purchased from ABX (advanced biochemical compounds) and were used without further purification. ^18^O-H_2_O was purchased from Huayi isotopes, China. Ethanol, Acetonitrile, and Water were HPLC grade from Merck. The extensive clinical and research application of PET in the past few years has stimulated a great interest in development of fully automated systems for ^18^FDG synthesis.

*Automated Radiosynthesis of* ^18^FDG

No carrier added aqueous fluoride (^18^F^-^) ion was produced on a MiniTrace Cyclotron (GE healthcare) by the irradiation of ^18^O-H_2_O via the ^18^O(p,n)^18^F nuclear reaction in a target chamber (7). The enriched water ^18^O-H_2_O (98% in ^18^O) irradiated with protons of 9.6 MeV for 2 h. The fully automated radiosynthesis of ^18^FDG was performed in a commercially available modular synthesis system (TRACERlab MX_FDG_ GE Healthcare) with a disposable ready-for-use cassette and reagent kit from ABX (ABX, advanced biochemical compounds, Germany). The cassette and reagent kit contain the Sep-Pak cartridges and chemicals required for ^18^FDG synthesis. The reagent kit includes: eluent solution, acetonitrile, mannose triflate precursor, ethanol, sodium hydroxide solution, buffer solution, and sterile water for injection. The resulting ^18^F ions from cyclotron were transferred to TracerLab MX_FDG_ synthesis module and were separated from enriched water using a quaternary ammonium anion exchange column (Sep Pak light accell plus QMA, waters), followed by elution from cartridge to reaction vessel using eluent solution (22 mg kryptofix, 7 mg potassium carbonate, 300 µL extrapure acetonitrile, 300 µL pure water). To prepare nucleophilic substitution conditions, the reaction mixture has to be dried by azeotropic distillation of the water with acetonitrile followed by evaporation under vacuum to make sure no water is left. The evaporation was carried out at 95 °C under nitrogen flow and vacuum. In next step, 25 mg mannose triflate precursor (1,3,4,6-tetra-O-acetyl-2-O-trifluoromethansulphonyl-b-D-mannopyranose, pharmaceutical grade) dissolved in high purity acetonitrile, was added to reaction vessel. Under the presence of kryptofix, triflate anion was replaced by ^18^F ion (SN2 reaction at 85 °C) and 2-fluoro-1,3,4,6-tetra-O-acetyl-D-glucose (ACY-^18^FDG) was formed (8). The reaction mixture was diluted with sterile water and transferred to reverse phase cartridge (Sep Pak plus tC18, Waters). ACY-^18^FDG was adsorbed on cartridge and polar by products (solvents, unreacted ^18^F ions, kryptofix) were removed by rinsing with water to waste bottle. To remove all protective groups, 750 µL sodium hydroxide 2N was applied to tC18 cartridge. The base hydrolysis happened at room temperature on column surface. The ^18^FDG was eluted with water from cartridge into buffer solution (5mL citrate buffer + 1mL HCl 2N) while un-hydrolyzed or partially hydrolyzed 1,3,4,6 acetyl protected compounds remained on the tC18 cartridge. The neutralized ^18^FDG solution was purified by passing through a second tC18 cartridge followed by passing through an Alumina N cartridge (Sep-pak plus Alumina N, waters) to retain partially hydrolyzed compounds, non-polar by products, un-reacted ^18^F ions, Na+ anions, and kryptofix. The product was sterilized by passing through a 0.22 µM sterile membrane filter. It is dispensed in syringes (kit Gemini B) using an automated system for the dispensing of ^18^FDG (Theodorico, Comecer). ^18^FDG sample is diluted 20 times with normal saline before QC tests and results are always corrected considering the dilution factor. The whole synthesis takes about 30 min. The resulting ^18^FDG solution (about 16 mL) is clear, colorless, neutral and isotonic. It is ready for Quality Control. The un-decay corrected yields of ^18^FDG is 50 ± 5% at the end of synthesis.

^18^ *FDG Quality Control*

PET drug producers have a quality control arrangement that is responsible for overseeing the production operations to ensure that each PET drug meets the safety requirements and has the identity and strength while adhering to the standards set for quality and purity characteristics (9, 10). The quality requirements of ^18^FDG are set out in various pharmacopoeia including USP (11), BP (12), EP (13), etc. The quality control of ^18^FDG is performed based on European Pharmacopeia 7.0. Basic requirements include appearance, radioactivity assay, pH, radionuclidic identity and purity, radiochemical identity and purity, chemical purity and microbiological purity.

*Appearance*

Appearance of ^18^FDG solution was determined by visual inspection under lead glass. 

*Radioactivity assay*

The radioactivity of QC sample was measured (A_1_) using Atomlab 500 dose calibrator (Biodex, USA) and compared with the radioactivity written on sample (A_0_) and calculated decay corrected (A_cal_) using the formula %RSD = [(A_cal_-A_1_)÷ A_cal_] × 100.

*pH*

Measurement of pH is one of the requirements before the release of the final product; this may be done using either a pH meter or with pH paper. The latter method is preferred because a smaller test sample is required. An aliquot of QC sample was dropped on indicator pH paper 0-14 (Universal indicator, Merck). 

*Radionuclidic identity and purity*

Radionuclidic identity and purity are evaluated by gamma-ray spectrometry and Half-life determination.

*Half-life determination: *An aliquot from QC sample was taken and the radioactivity was measured using Atomlab 500 dose calibrator every 10 min for 30 min under the same measuring or geometrical conditions. The half-life was determined using the formula t_1/2 _= (0.693 × t) ÷ ln(A_0_/A_t_) where: t_1/2_ (half-life), t (time interval in minutes), A_0_ (initial activity), A_t_ (activity measured after 10 min). 

*Gamma spectrum:* The gamma spectrum of an aliquot from QC sample was determined using Gamma Spectrometer (Multichannel Analyzer, BERTHOLD LB2045, USA).

*Radiochemical purity and identity*

*TLC:* About 2 µL of QC sample, and reference solution [a solution of 30 mg of 1,2,3,4-tetra-O-acetyl-β-D-glucopyranose (ABX) and 20 mg of gluocose (ABX) in 1 mL of water] were applied on silica gel plate (TLC silica gel 60 F_254_, Merck) with water:acetonitrile (5:95) as mobile phase over a path of 8 cm. The distribution of radioactivity was determined using TLC Scanner Mini-Scan (MS.1000, Bioscan) equipped with flow count (B-FC-1000, Bioscan) and gamma detector (MS3200, Bioscan). The plate was immersed in a 75 g/L solution of sulfuric acid in methanol and dried at 150 °C for chemical impurities. 

*HPLC:* 20 µL of reference solutions (a), (b), and QC sample were injected separately using HPLC (Agilent 1260, USA) equipped with flow count Radio-HPLC detector system (B-FC-1000, FC-3300, Bioscan) and pulse amperometric detector (RID), column (anion exchange resine, 0.25 m, 4.0 mm, 10 µM), mobile phase (0.1 N NaOH), flow rate (1 mL/min), run time (30 min). Reference solution (a): [FDG standard (ABX) in water 0.167 mg/mL], reference solution (c) [solution of FDM standard (ABX) 0.25 mg/mL and FDG standard (ABX) 0.083 mg/mL in water]. 

*Chemical purity*

Chemical contaminants may arise from procedures employed in the synthesis of ^18^FDG. These include residual organic solvents (such as acetonitrile and ethanol), catalysts (including aminopolyether), reagents and by-products, such as cold FDG, FDM, and glucose, depending on the method applied for the synthesis of ^18^FDG. 

Aminopolyether *(Kryptofix):* TLC silica gel plate for aminopolyether test was prepared by immersing the silica gel plate in iodoplatinate solution for 5-10 s and drying at room temperature for 12 h. 2.5 µL of QC sample, water, and reference solution were applied on silica gel plate. The spots were detected visually 1 min after application. 

Iodoplatinate solution [3 mL 10% W/V chloroplatinic acid (IV) (Merck) + 97 mL water + 100 mL 6% W/V KI (Merck)] 

*Solvents residues: *Residual solvents in the final solution (acetonitrile, ethanol) can be identified and quantified with a gas chromatograph (GC). A GC instrument should be equipped with a flame ionization detector (FID) and an appropriate column (packed column or capillary column) for the analysis of residual solvents 1 µL of reference solution [a solution of ethanol 0.167 mg/mL and acetonitrile 0.014 mg/mL in water] and QC sample were injected separately into GC (Agilent 7890) equipped with FID, agilent 125-7032 DB WAX 230 °C max megabor, 30.0 m, 0.53 mm, film thickness 1 µM, analysis time 6.3 min, FID temperature 300 C, oven temperature 200 °C. Reference solution was injected 2 times before and 1 time after QC sample injection. 

*Microbiological purity*

The microbiological purity of the final ^18^FDG product is evaluated by using tests for bacterial endotoxin (BET) and sterility. 

Bacterial endotoxins are quantified by the chromogenic method, using a Portable Test System–PTS® (Endosafe). This device includes a pumping system, a portable spectrophotometer and embedded software to calculate sample data. A sample is inserted into the cartridges (Endosafe) and the device assures duplicate samples and positive product control testing.

Sterility test must be initiated within a reasonable period of time, allowing for radioactivity to decay. A sterility test entails incubation of a test sample with two different growth media (soybean casein digest medium and fluid thioglycolate).

## Results and Discussion

The change in the quality of ^18^FDG under the influence of environmental factors has been investigated as a factor of time. The same physicochemical and microbiological parameters evaluated in ^18^FDG quality control were applied in the stability study. The specifications for the final product were established to meet all the EP (13) requirements as shown on table 1. These specifications are used as acceptance criteria for ^18^FDG approval and they were taken as acceptance criteria for this study. ^18^FDG radioactive concentration was kept between 0.3 - 0.5 GBq/mL. The main goal of this study was to evaluate if ^18^F-FDG samples would comply with all specifications after 10 h. This interval was chosen because it is an upper bound for the period between the end of the synthesis and the patient injection time (including transport).

^18^FDG samples were withdrawn 0, 2, 4, 6, 8 and 10 h after the final product was synthesized. All quality control assays were performed in the first and last evaluated periods, i.e., 0 and 10 h after synthesis. In the other time intervals, only radionuclidic identity, half-life and radiochemical identity and purity parameters were evaluated. To test the quality of ^18^FDG samples were diluted twenty times for Appearance, pH, radionuclidic identity, half-life, radiochemical identity and purity, chemical purity, residual solvents, bacterial endotoxin test. The results were corrected considering the appropriate dilution factor.

Radionuclidic purity and identity are evaluated by gamma-ray spectrometry (Figures 1-2). The gamma spectrum of a test sample should show a major peak at 511 KeV, and a sum peak at 1022 KeV, depending on the geometry and detector efficiency. No less than 99% of gamma emissions should correspond to ^18^F. This test should be performed periodically.

Radiochemical identity was evaluated by thin layer chromatography performed in silica gel with a solvent system of acetonitrile and water (95:5). The retention factor of spots of both the ^18^FDG sample and FDG standard (ABX) were compared. Figures 3 and 4 that obtained from TLC Scanner show typical chromatograms of ^18^FDG samples obtained just after the synthesis and 10 h later, respectively at room temperature and (35 – 40 ºC).

Radiochemical purity is determined by monitoring the radioactivity signal from the detector coupled in series with HPLC. Figures 5 and 6 that obtained from HPLC show typical chromatograms of ^18^FDG samples obtained just after the synthesis and 10 h later, respectively at room temperature and (35 – 40 ºC). 

Half-life can be determined within acceptable limits using counting equipment, such as a dose calibrator or a well counter. This is achieved by measuring the radioactivity of the sample at two or more time points and then calculating the decay. In practical consideration to the short half-life of ^18^F and the need to release the product as soon as possible, a precisely measured count at two points within a 10 min interval is sufficient to determine the physical half-life of ^18^F.

Results for ^18^F-FDG assays in all evaluated time intervals at different conditions of temperature are shown in Tables 2 and 3. The results indicated that ^18^FDG was able to comply with the specifications (Table 1) up to 10 h after the end of the synthesis, regarding acceptance limits for each required assay.

## Conclusion

The samples of ^18^FDG which were assayed using the European Pharmacopoeia. 7 edition. Analytical methods for Fludeoxyglucose F-18 injection complied with all the specifications up to 10 h after the end of the synthesis. These findings suggest that ^18^FDG has physicochemical and microbiological stability up to 10 h, if stored at room temperature or higher temperatures.
